# *R*_2_ Relaxometry of SABRE-Hyperpolarized Substrates at a Low Magnetic Field

**DOI:** 10.1021/acs.analchem.3c02709

**Published:** 2023-11-06

**Authors:** Pierce Pham, Christian Hilty

**Affiliations:** Chemistry Department, Texas A&M University, College Station, Texas 77843, United States

## Abstract

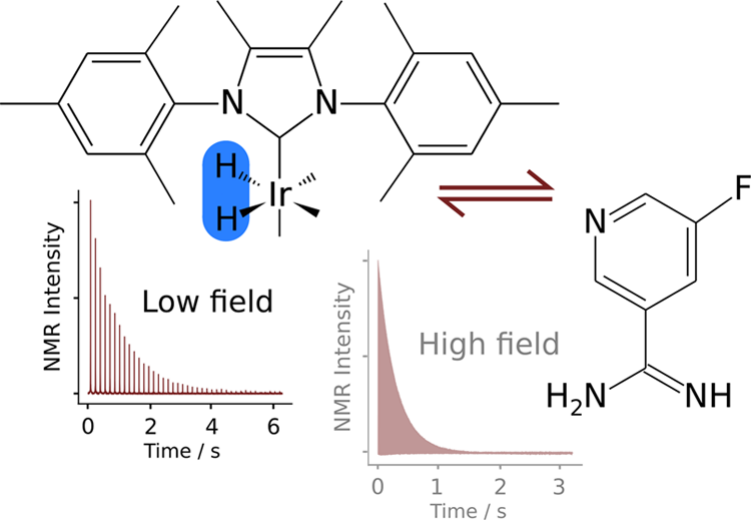

Nuclear magnetic resonance (NMR) relaxometry at a low magnetic field, in the milli-Tesla range or less, is enabled by signal enhancements through hyperpolarization. The parahydrogen-based method of signal amplification by reversible exchange (SABRE) provides large signals in a dilute liquid for the measurement of *R*_2_ relaxation using a single-scan Carr–Purcell–Meiboom–Gill (CPMG) experiment. A comparison of relaxation rates obtained at high and low fields indicates that an otherwise dominant contribution from chemical exchange is excluded in this low-field range. The SABRE process itself is based on exchange between the free and polarization transfer catalyst-bound forms of the substrate. At a high magnetic field of 9.4 T, typical conditions for producing hyperpolarization including 5 mM 5-fluoropyridine-3-carboximidamide as a substrate and 0.5 mM chloro(1,5-cyclooctadiene)[4,5-dimethyl-1,3-bis(2,4,6-trimethylphenyl)imidazol-2-ylidene]iridium(I) as a polarization transfer catalyst precursor resulted in an *R*_2_ relaxation rate as high as 3.38 s^–1^. This relaxation was reduced to 1.19 s^–1^ at 0.85 mT. A quantitative analysis of relaxation rates and line shapes indicates that milli-Tesla or lower magnetic fields are required to eliminate the exchange contribution. At this magnetic field strength, *R*_2_ relaxation rates are indicative primarily of molecular properties. *R*_2_ relaxometry may be used for investigating molecular interactions and dynamics. The SABRE hyperpolarization, which provides signal enhancements without requiring a high magnetic field or large instrumentation, is ideally suited to enable these applications.

## Introduction

Spin–spin (*R*_2_) relaxation is a highly sensitive parameter for characterizing molecular interactions by NMR. The dependence of *R*_2_ on molecular motions is well-known, increasing by several orders of magnitude if the rotational correlation time of the molecule changes from picoseconds to the range of nanoseconds. This parameter is useful for characterizing numerous chemical systems where an interaction results in a change in the effective rotational correlation time. Measurable chemical processes include, but are not limited to, the binding of ligands to proteins,^1−3^ interactions of adsorbents with surfaces,^4^ host–guest interactions,^5^ and molecules binding to or inserting in biological membranes.^6^*R*_2_ relaxation parameters measured with or without hyperpolarization are indicative of numerous types of interactions of small molecules with supramolecular complexes, nanoparticles and particle surfaces, or pores. In the gas phase, relaxation parameters of hyperpolarized xenon indicate adsorption in micro- and mesoporous materials or molecular cages.^7^ In liquids, the *R*_1_ and *R*_2_ relaxation rates are characteristic of the absorption of molecules in pores. The *R*_2_/*R*_1_ ratio can be used to characterize the interactions of solvent molecules with surfaces, with implications in numerous chemical processes such as heterogeneous catalysis.^4^ Molecular interactions over longer distances can be measured through paramagnetic relaxation enhancement, whereby paramagnetic labels introduced in a molecular species cause faster relaxation rates upon binding.^8^

Spin relaxation measurements can be significantly accelerated by using hyperpolarization. The signal enhancement provided by these methods generally permits the measurement of these parameters in a single scan. Dissolution dynamic nuclear polarization (D-DNP) has been widely applied to provide hyperpolarization in liquid-state NMR.^9^ D-DNP was shown to enable the detection of ligands binding to proteins at the micromolar level.^10,11^ Because a bound ligand assumes the large rotational correlation time of the macromolecule, an observable change in *R*_2_ can occur even if the fraction of the bound ligand is on the level of percent or less. The *R*_2_ relaxation rate can be used to detect the presence of the binding interaction, and in competition experiments measure the binding affinity to the protein.^1,2,12^

As an alternative, our group has recently proposed the use of parahydrogen for elucidating protein–ligand interactions.^1^ Parahydrogen-based hyperpolarization is in particular interesting for broader applications because of the ease with which the antiparallel para spin state of molecular hydrogen can be enriched at low temperatures.^13^ The signal amplification by reversible exchange is a parahydrogen-induced polarization method that converts this spin order into the polarization of a substrate molecule by interaction with a polarization transfer catalyst (PTC). Typical PTCs are iridium catalysts that can simultaneously bind parahydrogen and ligands, such as nitrogen heterocycles, amines, and carboxylic acids, to form octahedral structures.^14−16^ The ligands that bind in the equatorial positions, trans to iridium hydrides, are exchanged and enhanced most effectively in their NMR signal.^17^ The SABRE process depends on a relationship between the coupling constants in the polarization transfer complex and the frequency difference between the hydride hydrogen and the nucleus to be polarized. This dependence explains the optimal magnetic fields for the SABRE effect to occur, which are in the milli-Tesla range for ^1^H and in the micro-Tesla range for other nuclei such as ^15^N.^18^ Hyperpolarization produced by this means lends itself for combination with NMR detection at a low magnetic field, without superconducting or permanent magnets.^19^ It can also enable biomolecular applications such as the study of ligand binding under these conditions, which we recently demonstrated using ^19^F detection.^20^ Hyperpolarization provides a decisive signal enhancement for these applications, as illustrated in the following. A typical spin polarization level of 1% from SABRE can be compared to prepolarization at a magnetic field of 1 T and shuttling into the low-field magnet while retaining approximately 30% of the original polarization. Under these conditions, a 1-T prepolarized molecule of 1 M concentration results in a signal-to-noise ratio similar to that of a SABRE-hyperpolarized molecule at a concentration of ∼100 μM.

When used for the identification of protein binding, the hyperpolarized substrate should be a ligand of the protein of interest. The identification of ligand binding relies on an observed difference in *R*_2_ relaxation, which in the case of fast exchange between the free and bound forms of the ligand is the average of the respective relaxation rates. If the exchange between free and bound forms occurs on an intermediate time scale, then an additional contribution to the observed *R*_2_ relaxation time constant results. This change forms the basis of *R*_2_ relaxation dispersion measurements,^21^ i.e., the determination of *R*_2_ relaxation as a function of the refocusing time in a train of spin echoes. The*R*_2_ relaxation dispersion can be used to find the lifetime of the complex and may also provide information on the structure of the bound ligand.^3^

At the same time, the fact that *R*_2_ relaxation is subject to multiple contributions can complicate its use for the analysis of the underlying molecular properties. The effect of chemical exchange or reaction kinetics^22^ can mask the dependence on intra- and intermolecular motions deriving from the spectral densities of motions in relation to the NMR frequency. The exchange contribution is a function of the frequency difference of the spins in the exchanging molecular sites. In this paper, we demonstrate that low-field NMR with insignificant chemical shift differences presents a promising alternative for *R*_2_ relaxation measurements in the absence of exchange contributions. We employ nuclear spin hyperpolarization by the SABRE method to make NMR observation of dilute species feasible. At a measurement field of 0.85 mT, this hyperpolarization results in a calculated signal gain of >10^6^, whereas the Boltzmann spin polarization is generally insufficient for observing signals. We characterize exchange rates and the corresponding relaxation contributions that are due to the binding of the hyperpolarizable substrate to the polarization transfer catalyst and compare measurements at high and low magnetic fields with calculated line shapes.

## Experimental Section

**Figure 1:**
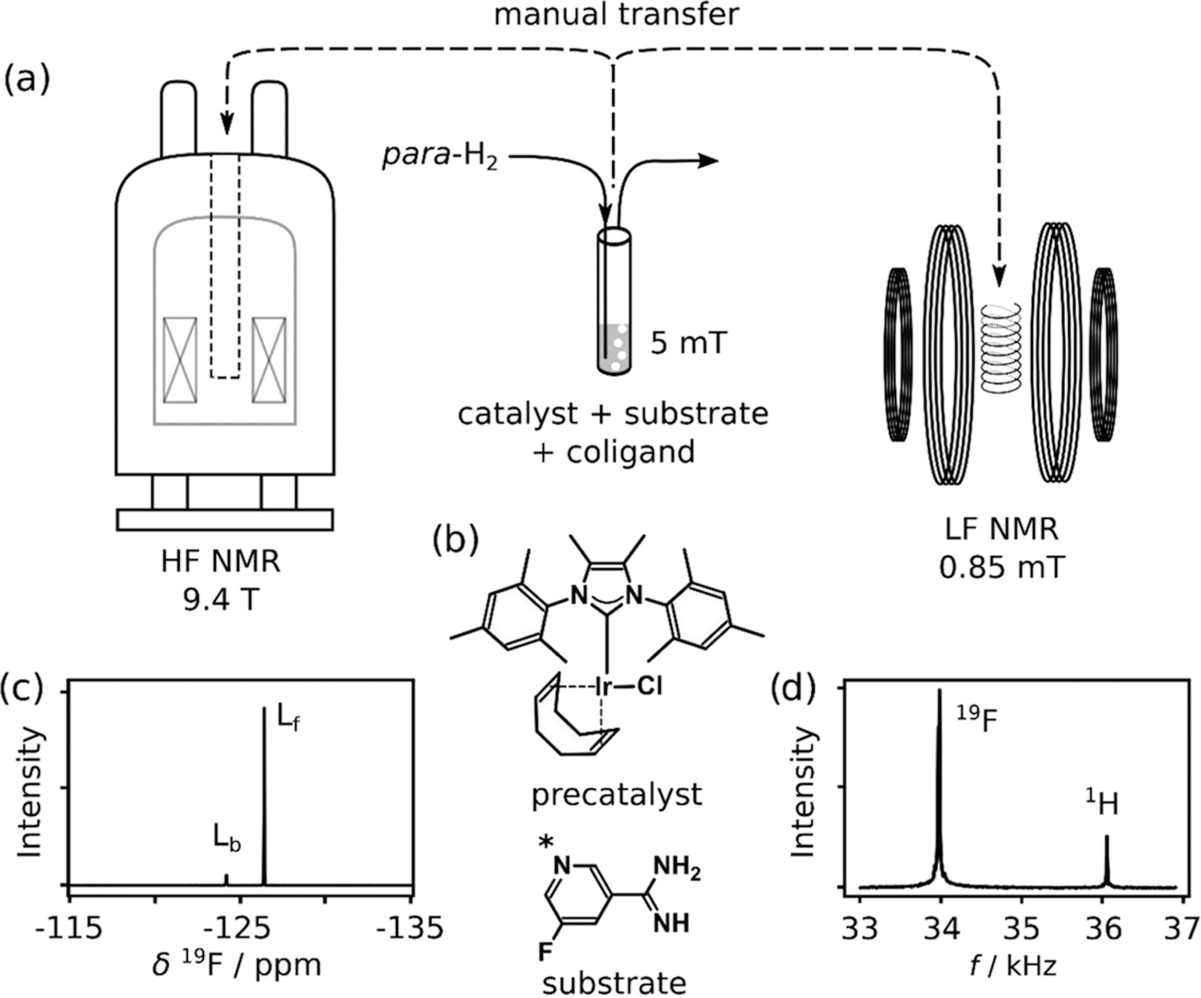
(a) Illustration of SABRE hyperpolarization and *R*_2_ relaxation measurements. SABRE samples were hyperpolarized at 5 mT and then manually transferred to either a low-field (0.85 mT) or high-field (9.4 T) NMR spectrometer for measurements. (b) Structures of the precatalyst and ligand. The asterisk indicates the site of binding of this ligand to iridium. (c) High-field ^19^F NMR spectrum of a SABRE-hyperpolarized sample. The signals from the catalyst-bound (L_b_) and free substrate (L_f_) are indicated (1 ppm = 376.6 Hz). (d) Low-field frequency spectrum of a SABRE-hyperpolarized sample with signals of ^19^F and ^1^H spins indicated.

The *R*_2_ relaxation rates of ^19^F spins in SABRE-hyperpolarized 5-fluoropyridine-3-carboximidamide hydrochloride were measured at high and low magnetic fields (Figure 1a). The samples consisted of 0.5 mM chloro(1,5-cyclooctadiene)[4,5-dimethyl-1,3-bis(2,4,6-trimethylphenyl)imidazol-2-ylidene]iridium(I) (Strem, Newburyport, MA) as the precatalyst, 5 mM 5-fluoropyridine-3-carboximidamide hydrochloride as the substrate to be hyperpolarized, and 5 mM dimethyl sulfoxide (Alfa Aesar, Ward Hill, MA) as a coligand for the polarization transfer catalyst (Figure 1b).^23^ For samples with higher substrate concentrations, a 15 mM substrate and 15 mM coligand were used. The solvent was methanol (Fisher Scientific, Hampton, NH). To synthesize 5-fluoropyridine-3-carboximidamide hydrochloride, 5-fluoropyridine-3-carbonitrile (Ambeed, Arlington Heights, IL) was reacted with sodium methoxide (Alfa Aesar, Ward Hill, MA) and subsequently with ammonium chloride (Alfa Aesar), followed by purification through crystallization.^20,24^

For the NMR experiments, the samples were pressurized with 120 psi hydrogen gas for 5 min to activate the precatalyst. This pressure was maintained throughout the entire experiment. The parahydrogen was enriched at a temperature of 29 K by using a cryogenic system (Advanced Research Systems, Macungie, PA). To achieve SABRE hyperpolarization, the parahydrogen gas was bubbled through the samples with a flow rate of 0.2 slpm for 20 s. During this time, the samples were placed in a solenoidal electromagnet with a magnetic field of 5 mT. Immediately after bubbling, the samples were manually transferred to either a low-field (0.85 mT) NMR spectrometer or a high-field (9.4 T) NMR spectrometer (Bruker Biospin, Billerica, MA). The time for the sample transfer was 4 s. In the NMR spectrometers, *R*_2_ relaxation rates were measured with Carr–Purcell–Meiboom–Gill pulse trains that included π pulses for refocusing separated by a duration of 2τ_CPMG_. The low-field experiments were performed in a lab-made spectrometer that was previously described.^25^ A data acquisition board (PCIe-6259 or PCIe-6363, NI, Austin, TX) was used to generate NMR pulses and simultaneously acquire signals. The sampling rate for both tasks was 800 kHz. CPMG pulse trains included a series of 40 refocusing pulses with τ_CPMG_ = 80 ms or a series of 20 refocusing pulses with τ_CPMG_ = 160 ms. In either case, the central portions of the signal between pulses of 128 ms duration were extracted and Fourier transformed without applying any window functions. In the resulting spectra, signals at 34 kHz were integrated and fitted to *R*_2_ relaxation rates. For high-field experiments, a 400 MHz NMR spectrometer equipped with a board-band observe (BBO) probe (Bruker Biospin) was used. CPMG pulse trains included a series of 6144 π pulses with τ_CPMG_ = 0.8 ms, and 64 complex points were acquired per echo. The π/2 pulse length was 0.575 ms (γ*B*_1_ = 435 Hz). After the Fourier transform of each echo, peaks were integrated and fitted.

The ligand exchange rates were measured with a series of EXSY-type experiments that were conducted at the high field. The NMR pulse sequence for these experiments included a ^19^F selective excitation of free ligands, various mixing times in the range of 10–80 ms, and a hard π/2 pulse before data acquisition. The ratios of the bound and free ligands with varied mixing times were fitted by a kinetic model (Supporting Information).

## Results and Discussion

Spectra of 5-fluoropyridine-3-carboximidamide hydrochloride, measured after SABRE hyperpolarization at high and low fields, are shown in Figure 1c,d, respectively. The high-field spectrum was measured with the NMR probe tuned to the ^19^F frequency. From this sample, separate signals for the catalyst-bound and free substrate molecules are observed at chemical shifts of −124.16 and −126.36 ppm, respectively. As evidenced by the stronger signal for the free substrate, this species is in excess, a condition that leads to efficient SABRE hyperpolarization. The relative signal intensity of the catalyst-bound substrate is further reduced by a more substantial exchange broadening, the details of which are discussed in the following. Only one species of bound ligand is observable and contributes to further analysis; other bound species, if existing, would have a negligible influence. The usage of sulfoxides as coligands stabilizes SABRE-active catalysts with weakly binding ligands.^23^ From the observed free and bound fractions, the ligand-bound catalyst most likely is (NHC)IrH_2_(DMSO)_2_L or (NHC)IrH_2_Cl(DMSO)L, and the exact identity of the catalyst will not be considered in further analysis of *R*_2_ relaxation rates.

In the low-field NMR spectrum of the SABRE-hyperpolarized sample shown in Figure 1d, the two observable signals at 34.0 and 36.2 kHz correspond to ^19^F and ^1^H spins. Only one signal from each nucleus can be observed because chemical shifts are not resolved in the 0.85 mT measurement field. The ^19^F signal is larger than that of ^1^H foremost because the excitation pulse and tuning of the detection coil were optimized for this frequency. The comparison of Figure 1c,d further illustrates that in the absence of chemical shift resolution, alternative observables need to be used for chemical identification. These include *J*-coupling constants, which can be resolved with high precision at a low field^26^ and can be measured at zero field.^27^ In other applications, a molecule such as the 5-fluoropyridine-3-carboximidamide used here can be designed as a probe to include a heteronuclear label.^20^ Apart from ^19^F,^28^ different modalities of SABRE can be used to hyperpolarize lower-frequency nuclei such as ^15^N.^29^

**Figure 2:**
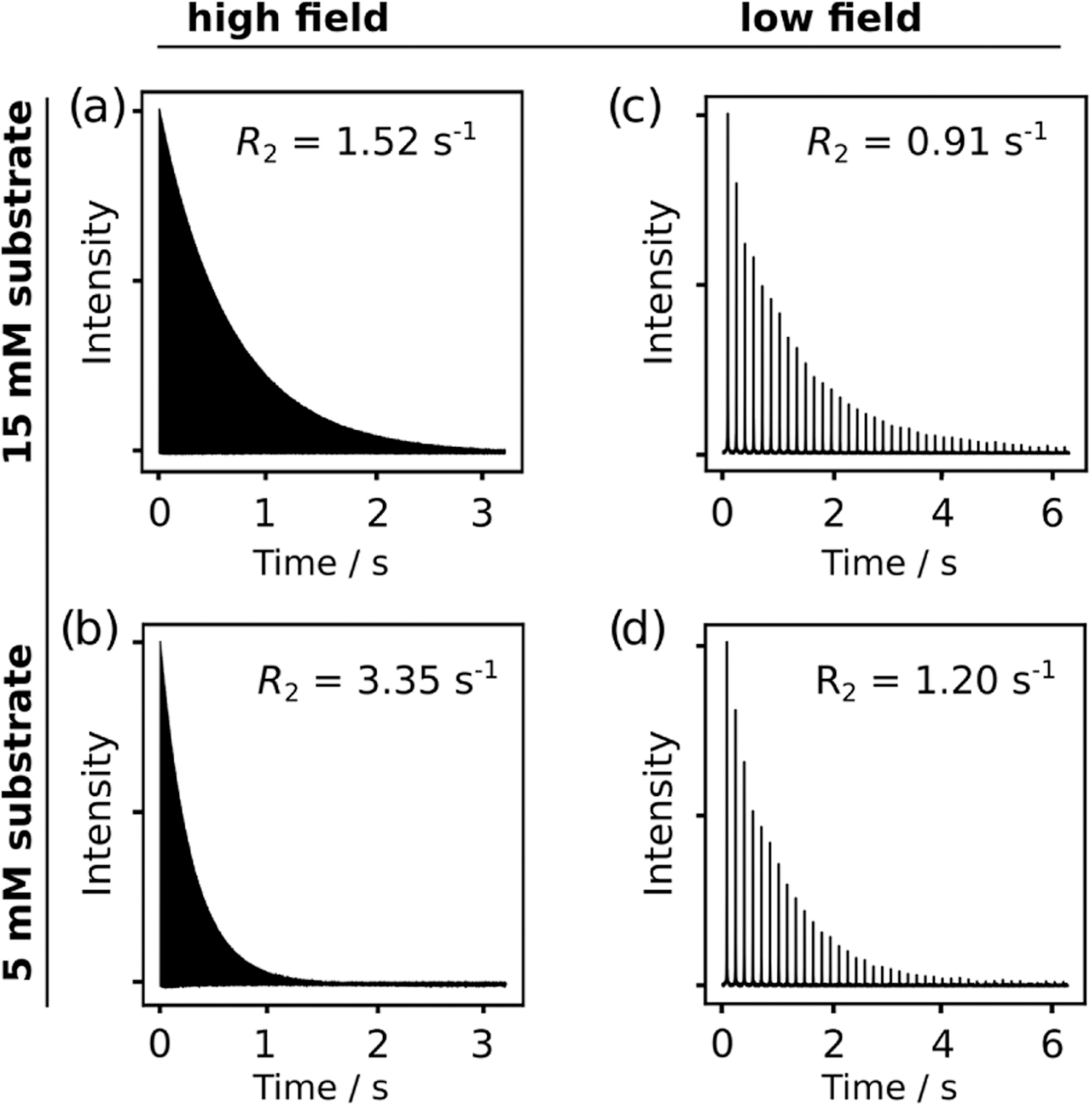
Time-dependent ^19^F NMR signals of the SABRE-hyperpolarized substrate and fitting results of *R*_2_ relaxation rates. Each plot contains a series of signals from Fourier transformed spin echoes of SABRE-hyperpolarized substrates. The left panels show the first 2000 spectra obtained at the high field, and the right panels show the entire 40 spectra obtained at the low field. The top and bottom panels are the results for the samples with high and low concentrations of the substrate, respectively. The complete details of data acquisition and fitting are described in the Experimental Section, and the complete fitting results for all measurements are shown in the Supporting Information.

The *R*_2_ relaxation rates of the peak from the free substrate at the high field, and of the overall ^19^F signal at the low field were measured using CPMG experiments. The experiments were performed at two different substrate concentrations of 5 and 15 mM. Representative data sets are shown in Figure 2. In these graphs, the corresponding signals from the Fourier transform of each spin echo are stacked, resulting in an observable signal decay due to *R*_2_ relaxation.

**Figure 3:**
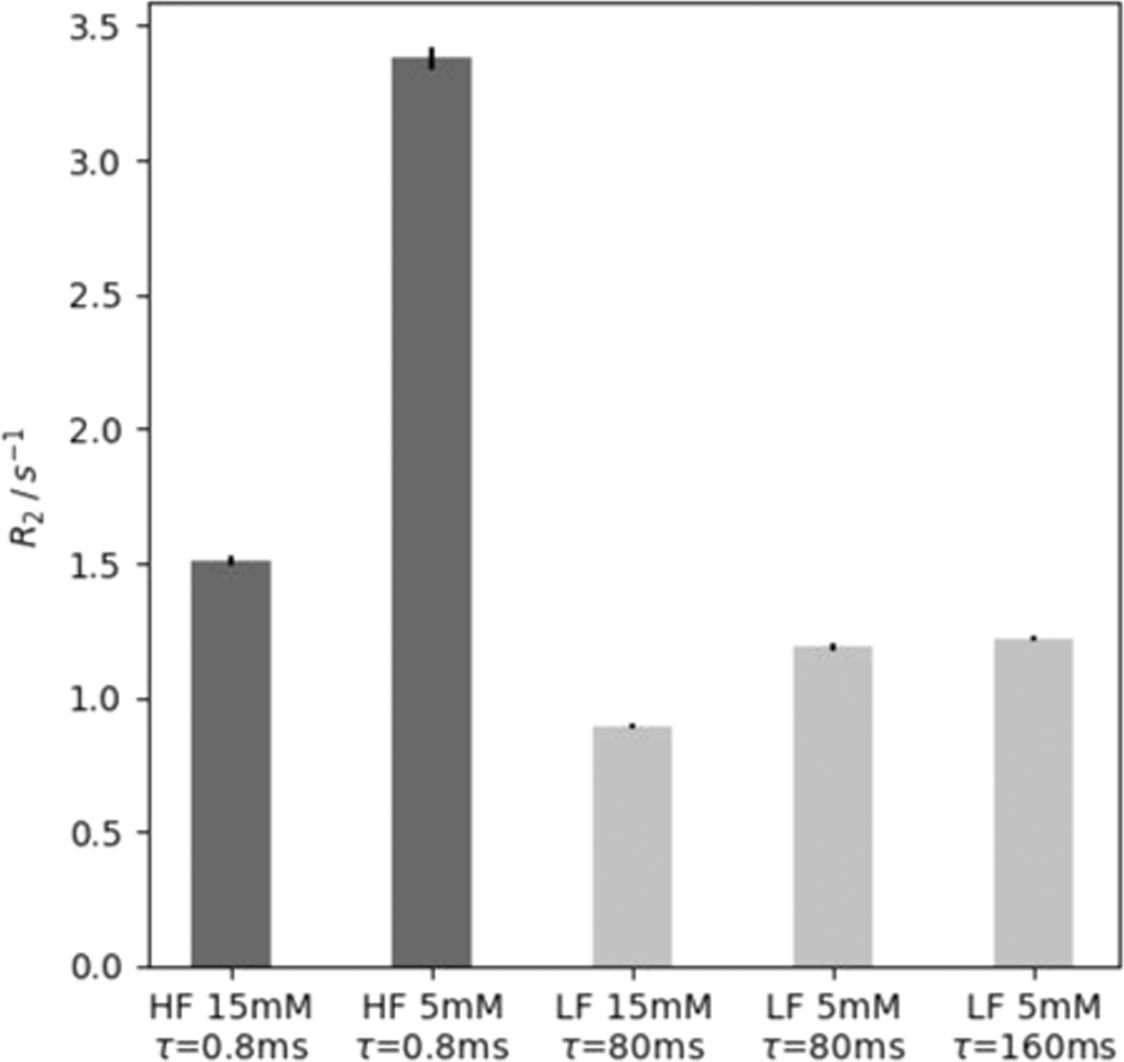
Bar graph comparing the measured *R*_2_ relaxation rates. The labels indicate the field strength, high field at 9.4 T (HF), or low field at 0.85 mT (LF), the concentration of the substrate and DMSO at 15 or 5 mM, and the CPMG refocusing delay τ. HF 15 mM: 1.52 ± 0.02 s^–1^; HF 5 mM: 3.38 ± 0.04 s^–1^; LF: 0.895 ± 0.009 s^–1^; LF 5 mM (80 ms): 1.19 ± 0.01 s^–1^; LF 5 mM (160 ms): 1.22 ± 0.01 s^–1^ (Figures S2–S6).

The average results for *R*_2_ relaxation rates fitted from several measurements under each condition (Supporting Information) are summarized in Figure 3. It is evident that at the high field, the relaxation rates are substantially larger than the *R*_2_ relaxation rate of the free substrate, which was determined without hyperpolarization as *R*_2,f_ = 0.50 ± 0.02 s^–1^ (Figure S1). The relaxation is faster for the sample at the lower substrate concentration because the fraction of the catalyst-bound substrate and the exchange broadening are larger.

At the high field, the measured *R*_2_ relaxation rates depend on the CPMG delay τ. A refocusing delay that is short compared to the exchange lifetime will result in refocusing of the exchange broadening, whereas *R*_2_ rates can include a significant exchange contribution when τ is longer. In other applications of SABRE-polarized molecules, the dependence of *R*_2_ on τ, i.e., the *R*_2_ relaxation dispersion,^21^ may be used to determine chemical exchange dynamics.

The observed relaxation rates at the low field are lower. Under this condition, the catalyst-bound and free species are not separately resolved, and exchange line broadening is eliminated. Consequently, the difference of rates between high and low concentrations is solely due to the averaging of the relaxation rates of free and catalyst-bound substrate, *R*_2,obs_ = *p*_b_*R*_2,b_ + (1 – *p*_b_)*R*_2,f_. The fractions of bound and free substrates under the two sample conditions were determined from signal integration in the high-field NMR spectra. These integrations yielded the bound *p*_b_ = 0.11 ± 0.02 for the lower concentration and *p*_b_ = 0.038 ± 0.002 for the higher concentration. From these values, it is possible to directly determine the *R*_2,b_ = 4.8 ± 1.1 s^–1^ and *R*_2,f_ = 0.74 ± 0.05 s^–1^. These parameters are directly dependent on the molecular properties and independent of chemical exchange. The stated uncertainty ranges include experiment-to-experiment variations from three repetitions and two samples. The observed *R*_2_ rates of the free substrate are different between the measurement at the high field and the calculation at the low field. A cause for this difference could be the effect of diffusion and *B*_0_ inhomogeneity combined with longer τ_CPMG_, which can lead to an increase in the observed *R*_2_ rates, as well as experimental imperfections such as *B*_1_ inhomogeneity.

At a low field, it is possible to use a lower refocusing rate in the CPMG experiment because of the absence of the frequency difference of free and bound substrates that would need to be refocused. As stated above, only external contributions to relaxation, such as those arising from the magnetic field inhomogeneity and diffusion, need to be considered. The ability to measure relaxation rates with a longer echo time is further evidenced by the rightmost data set in Figure 3, which resulted in a comparable relaxation rate after doubling the τ_CPMG_ value. Similar values indicate an overall limited effect of diffusion and *B*_0_ inhomogeneity on the results of the experiment.

The differences in the observed relaxation rates can be explained quantitatively by considering the chemical exchange contributions. For this purpose, the exchange rates for the substrate binding to the catalyst were measured by using a series of exchange spectroscopy (EXSY) NMR experiments (Figure S7). The results show *k*_ex_ = 23.0 ± 4.0 and 24.6 ± 1.3 s^–1^ for the lower and higher concentrations of the substrate, respectively. The observed ^19^F-frequency differences (Δ*ν*) between the bound and free substrates are 828 ± 0.8 and 827 ± 1.2 Hz for the lower and higher concentrations of the substrate, respectively. In the following, the average frequency difference of 827 ± 1.1 Hz is used for the calculation. Because *k*_ex_ ≪ Δ*ν*, the chemical exchange of this substrate falls into the slow regime, and *R*_2,f,slow_ = *R*_2,f_ + *k*_a,app_. Here, *R*_2,f,slow_ designates the approximate *R*_2_ rate of the free substrate under the assumption of slow exchange.^30^ From the experimental data, *R*_2,f,slow_ = 3.02 ± 0.05 and 1.42 ± 0.04 s^–1^ for the lower and higher substrate concentrations. These calculated *R*_2,f,slow_ values match well with the observed *R*_2_ rates in Figure 3. Observed differences, of 10% or less, are most likely caused by additional error contributions, including those from the use of the simplified two-site exchange model and the possible contribution of the *R*_2_ relaxation rates of bound substrates.

**Figure 4:**
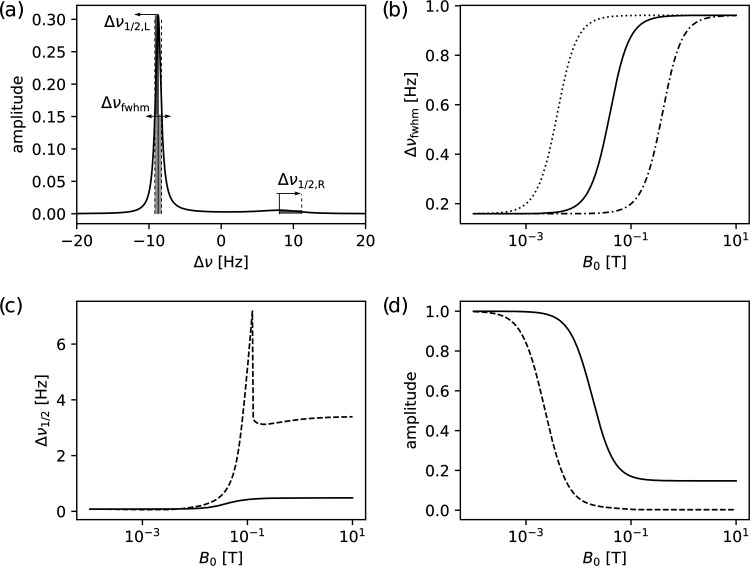
(a) Line shapes calculated with the parameters from the low-concentration experiment, for *B*_0_ = 0.2 T. The frequency of the “free substrate” is in the left half of the spectrum and of the “bound substrate” in the right half. (b) Dependence on *B*_0_ of the full width at half-maximum (fwhm) of the tallest peak, plotted for a frequency difference from the experiment (828 Hz; –), 10 times smaller (82.8 Hz; ···) and 10 times larger (8.28 kHz; – · –). (c) Solid line: Left-sided half-width of the signal in the left half of the spectrum. The width is indicated as ←Δν_1/2,L_ in panel (a) and would be associated with the free substrate in the limit of slow exchange. Dashed line: Right-sided half-width in the right half of the spectrum. This width is indicated as Δν_1/2,R_→ in (a) and would be associated with a bound substrate in the slow exchange limit. (d) Solid line: Maximum amplitude of the signal in the left half of the spectrum (“free substrate”). Dashed line: Maximum amplitude of the signal in the right half of the spectrum (“bound substrate”).

In the following, the field dependence of the exchange contribution to *R*_2_ is considered. Figure 4a illustrates the calculated line shape according to the solutions of the Bloch–McConnell equations, from ref (31), for an intermediate magnetic field of 0.2 T. It can be seen that the signal close to the frequency of the free substrate is tallest, whereas the signal near the frequency of the bound substrate is substantially exchange broadened. In Figure 4b–d, the *B*_0_ field dependence of different parameters of these signals is plotted. The line width of the taller signal, which can primarily be observed experimentally, is shown in Figure 4b. It is noted that for a Lorentzian line, corresponding to a single exponential decay in the time domain, *R*_2_ = πΔν_fwhm_. Therefore, the plotted line widths are in good agreement with the observed data from Figure 3. The line width of the observed signal transitions from a plateau corresponding to a broader shape in the high-field limit that includes the high-field data in Figure 3 close to 10^1^ T, to a narrower shape corresponding to the low-field experiments below 10^–3^ T. The width of the broader signal, represented in Figure 4c as the right half-width, becomes large in the transition region at an intermediate magnetic field strength before merging with the taller signal at a low field. Here, the right half-width is defined as the frequency difference starting from the signal maximum in the right half of the spectrum and moving to the right until the intensity is half. This definition would be equivalent to the half-width at half-maximum for a symmetric signal but results in meaningful values in all cases, specifically also when the peaks are coalescing. The same transition at the intermediate magnetic field strength is also observable when considering the maximum amplitude in the left and right halves of the spectrum (Figure 4d), which starts at a lower value for the broader signal at a high field, ultimately transitioning to the higher value as the two signals merge in the center of the spectrum. The last part of this change in the graph, near 10^–3^ T, is primarily due to the signal moving to the center of the spectrum, which causes the amplitude measured from the right half to increase. According to Figure 4b–d, to reach the narrowest line and thus be able to measure relaxation rates without exchange contribution, a field as low as in the milli-Tesla range is indeed required. Typical field strengths that are, for example, encountered in benchtop NMR spectrometers employing permanent magnets, in the 10^0^ T range, are still high fields in the context of this experiment. The specific cutoff for the experiment to be considered at the low field depends on the chemical shift difference of the signals and on the exchange rate. The former dependence is illustrated in Figure 4b, where the dashed and dash-dotted lines correspond to 10 times smaller and 10 times larger frequency differences.

The above data illustrate that the exchange contribution to *R*_2_ relaxation can be reduced to an, for all practical purposes, insignificant level by performing relaxometry experiments in the milli-Tesla magnetic field range. Other contributions to *R*_2_, such as due to the change in the correlation time upon binding to a macromolecule, larger particle, or surface, do not exhibit the same strong magnetic field dependence. Thus, milli-Tesla NMR would provide the ability to study these molecular interactions and properties through *R*_2_ relaxometry. While low-field NMR does not provide chemical shift resolution that may be used for the identification of chemical compounds, the absence of multiple signals results in an absence of the exchange contribution to *R*_2_. This property of low-field NMR can simplify the relaxation analysis and potentially reveal effects that would otherwise be obscured by the large line broadening. Additionally, we note that the absence of an observable chemical shift difference between the two exchanging forms of the molecule precludes the use of corresponding signal integrals to determine concentration fractions. Here, fractions *p*_b_ and (1 – *p*_b_) were determined from the high-field NMR spectra. However, it is alternatively possible to perform such measurements using low-field NMR data only, by titrating the concentration. The concentration fractions can then be determined by fitting the observed *R*_2_ values to the equation *R*_2,obs_ = *p*_b_*R*_2,b_ + (1 – *p*_b_)*R*_2,f_.

Since the nuclear spin population difference at thermal equilibrium in a milli-Tesla magnetic field is insufficient to obtain signals from dilute solutions, another means to prepolarize nuclear spins is required. Prepolarization may be achieved by placing the sample in a high magnetic field before the measurement. However, larger nuclear spin polarizations can generally be achieved using a hyperpolarization method. Here, the parahydrogen-derived SABRE method provided a signal enhancement of several hundred fold compared to the signal level obtained at 9.4 T. This translates into a signal improvement of more than a million fold at the low field and makes it possible to measure substrates at millimolar or lower concentrations.

SABRE is suitable for low-field NMR because of the relative ease with which parahydrogen can be produced. The SABRE effect itself is effective at a magnetic field in the milli-Tesla range or below. The SABRE mechanism relies on chemical exchange for binding to the polarization transfer catalyst. For the SABRE process to work efficiently, the affinity of the polarization transfer catalyst to the substrate needs to be tuned for the substrate to be in reversible exchange with an optimal exchange rate. This chemical exchange leads to the *R*_2_ relaxation described above at the high field, but is insignificant at the low magnetic field. The combination of SABRE hyperpolarization with a low-field NMR measurement therefore can provide an ideal solution for *R*_2_ relaxometry in compatible applications.

## Conclusions

Low-field NMR in the milli-Tesla magnetic field range or below eliminates the exchange contribution to the *R*_2_ relaxation. At the same time, parahydrogen polarization through the SABRE method provides a signal enhancement that can enable the measurement of dilute spins of interest at a low field. Although the SABRE method relies on chemical exchange in the binding to a polarization transfer catalyst, the line broadening effect of this process is also eliminated at a low field. These features of the SABRE-enhanced low-field NMR technique should facilitate the characterization of molecular dynamics, molecular interactions, surface properties, and porosity by *R*_2_ relaxometry.

## Supporting Information Available

Graphs of CPMG data of high-field experiments, CPMG data of low-field experiments, and exchange spectroscopy (PDF)
