# Supplementary material for: R2 Relaxometry of SABRE-Hyperpolarized Substrates at a Low Magnetic Field
Source: Anal Chem. 2023 Nov 6;95(46):16911–7. doi: 10.1021/acs.analchem.3c02709 (PMC10862376; doi:10.1021/acs.analchem.3c02709)
Supplement: Supplementary file 1 — ac3c02709_si_001.pdf [file ac3c02709_si_001.pdf]

Supporting Information

R_2 Relaxometry of SABRE Hyperpolarized Substrates at Low Magnetic Field

Pierce Pham and Christian Hilty*

Chemistry Department, Texas A&M University, College Station, TX 77843, USA
*email: chilty@tamu.edu

Table of Contents

CPMG Data of High-field Experiments........2
CPMG Data of Low-field Experiments........5
Exchange Spectroscopy........8

CPMG Data of High-field Experiments

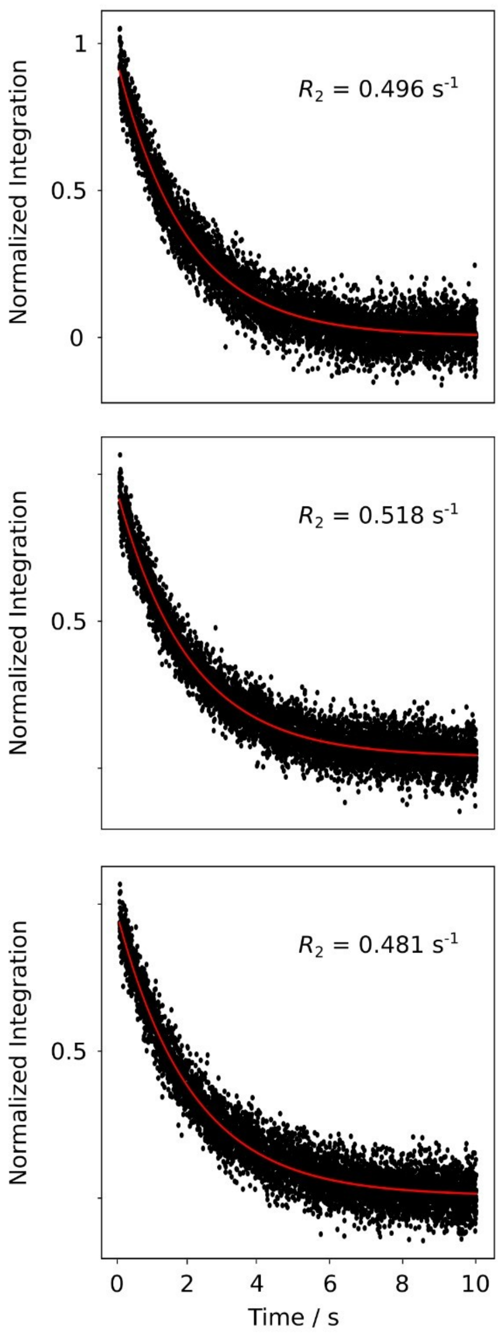

Figure S1: Fitting results of CPMG experiments at high fields (9.4 T), $R_2 = 0.50 \pm 0.02$ s^{-1}. Three samples consisted of 15 mM 5-fluoropyridine-3-carboximidamide hydrochloride in methanol. CPMG trains include 6144 π pulses and $\tau_{CPMG} = 0.8$ ms.

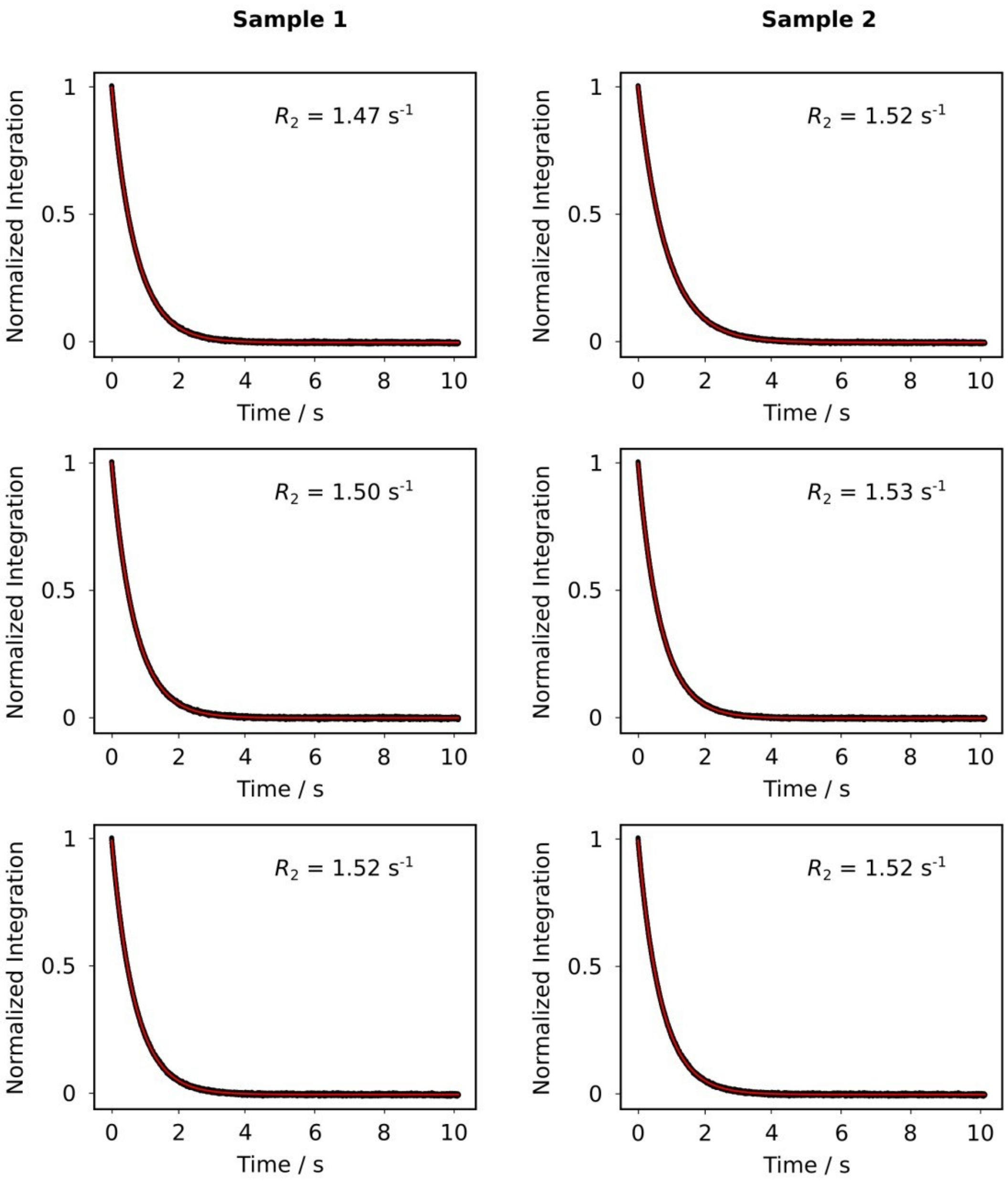

Figure S2: Fitting results of CPMG experiments at high fields (9.4 T), R_2 = 1.52 ± 0.02 s^{-1}. Two samples consisted of 0.5 mM precatalyst, 15 mM 5-fluoropyridine-3-carboximidamide hydrochloride, and 15 mM dimethyl sulfoxide in methanol. Three measurements were conducted per sample. CPMG trains include 6144 π pulses and τ_{CPMG} = 0.8 ms.

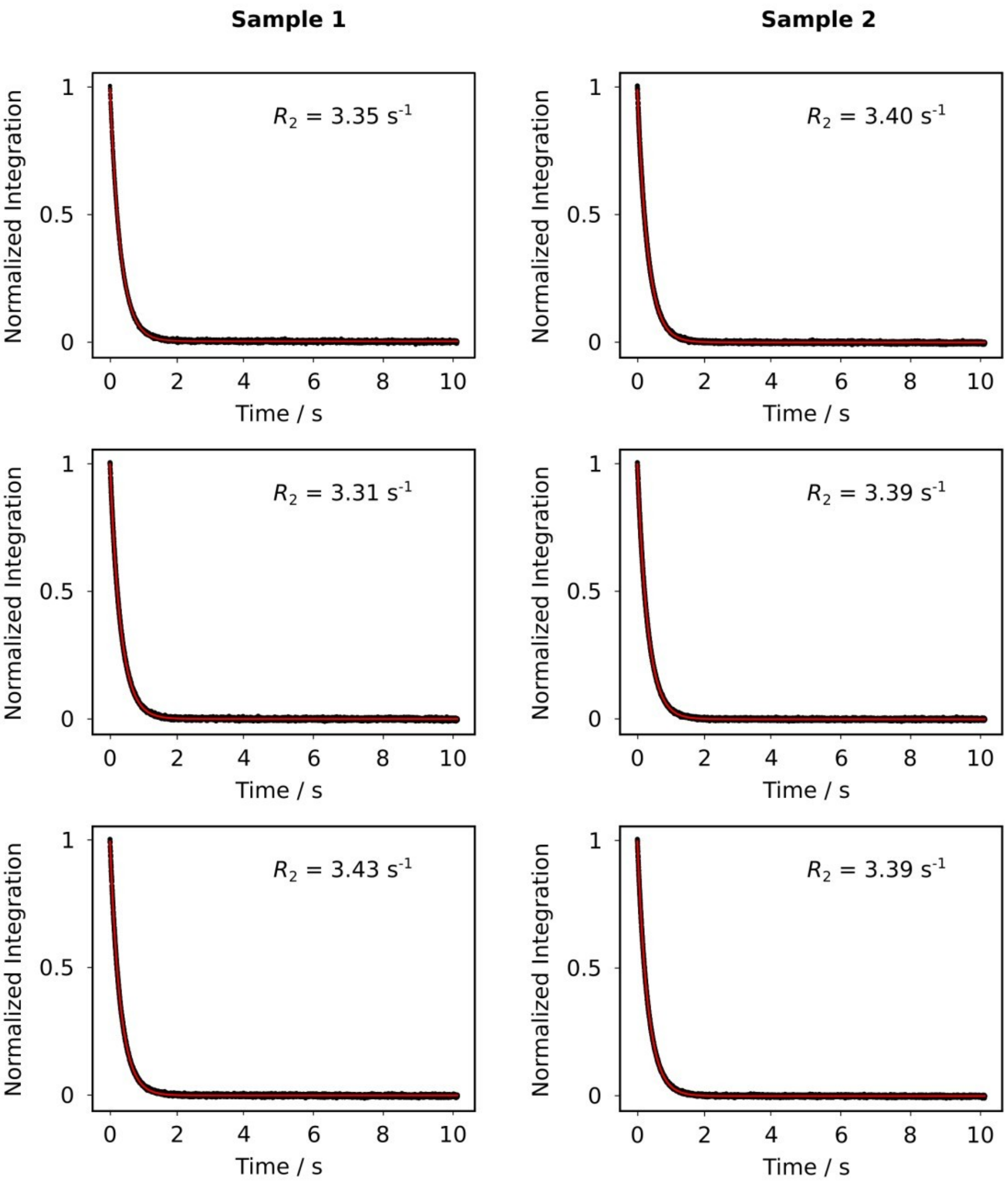

Figure S3: Fitting results of CPMG experiments at high fields (9.4 T), $R_2 = 3.38 \pm 0.04\ s^{-1}$. Two samples consisted of 0.5 mM precatalyst, 5 mM 5-fluoropyridine-3-carboximidamide hydrochloride, and 5 mM dimethyl sulfoxide in methanol. Three measurements were conducted per sample. CPMG trains include 6144 π pulses and τ_{CPMG} = 0.8 ms.

CPMG Data of Low-field Experiments

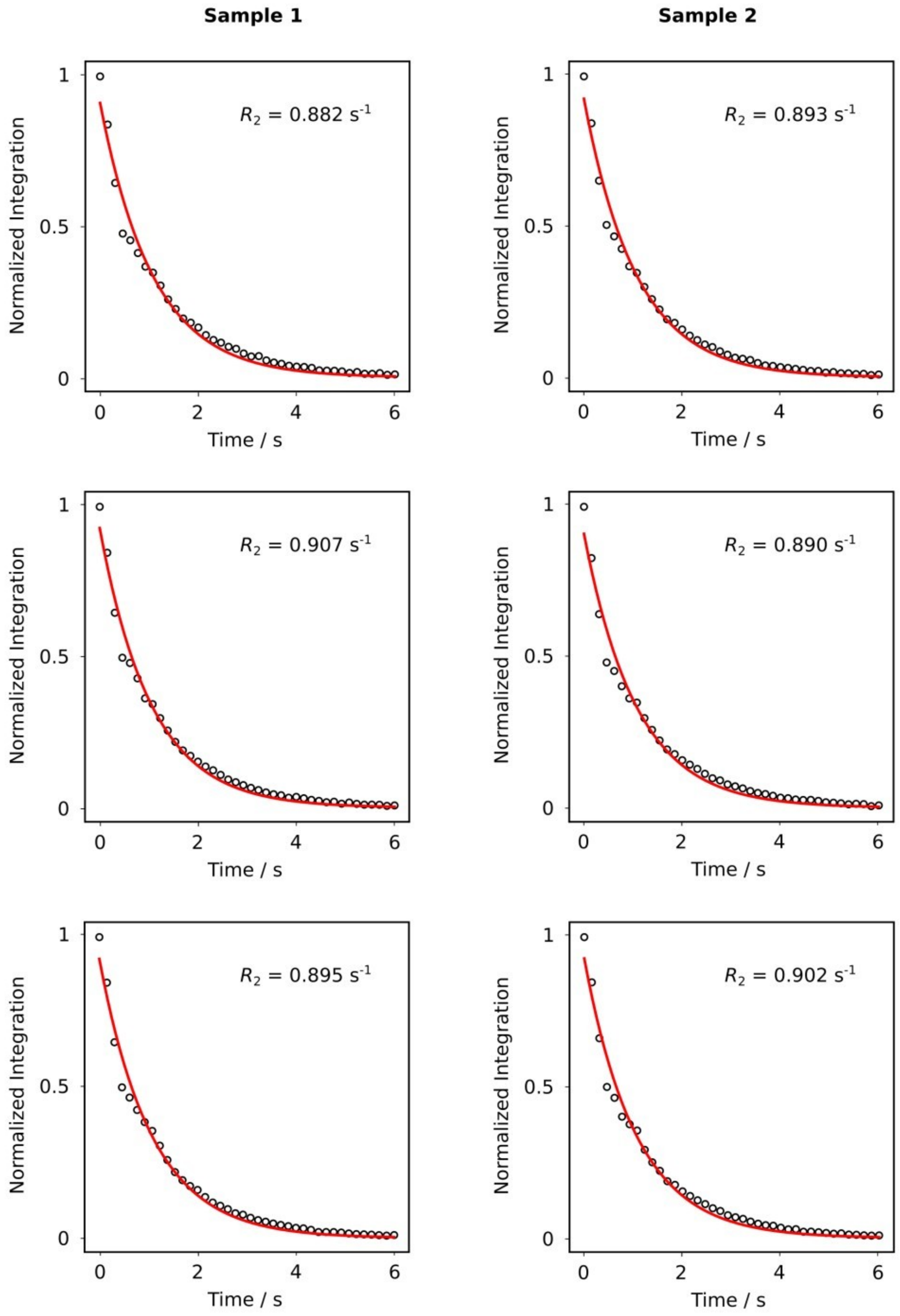

Figure S4: Fitting results of CPMG experiments at low fields (0.85 mT), R_2 = 0.895 ± 0.009 s^{-1}. Two samples consisted of 0.5 mM precatalyst, 15 mM 5-fluoropyridine-3-carboximidamide hydrochloride, and 15 mM dimethyl sulfoxide in methanol. Three measurements were conducted per sample. CPMG trains include 40 π pulses and τ_{CPMG} = 80 ms.

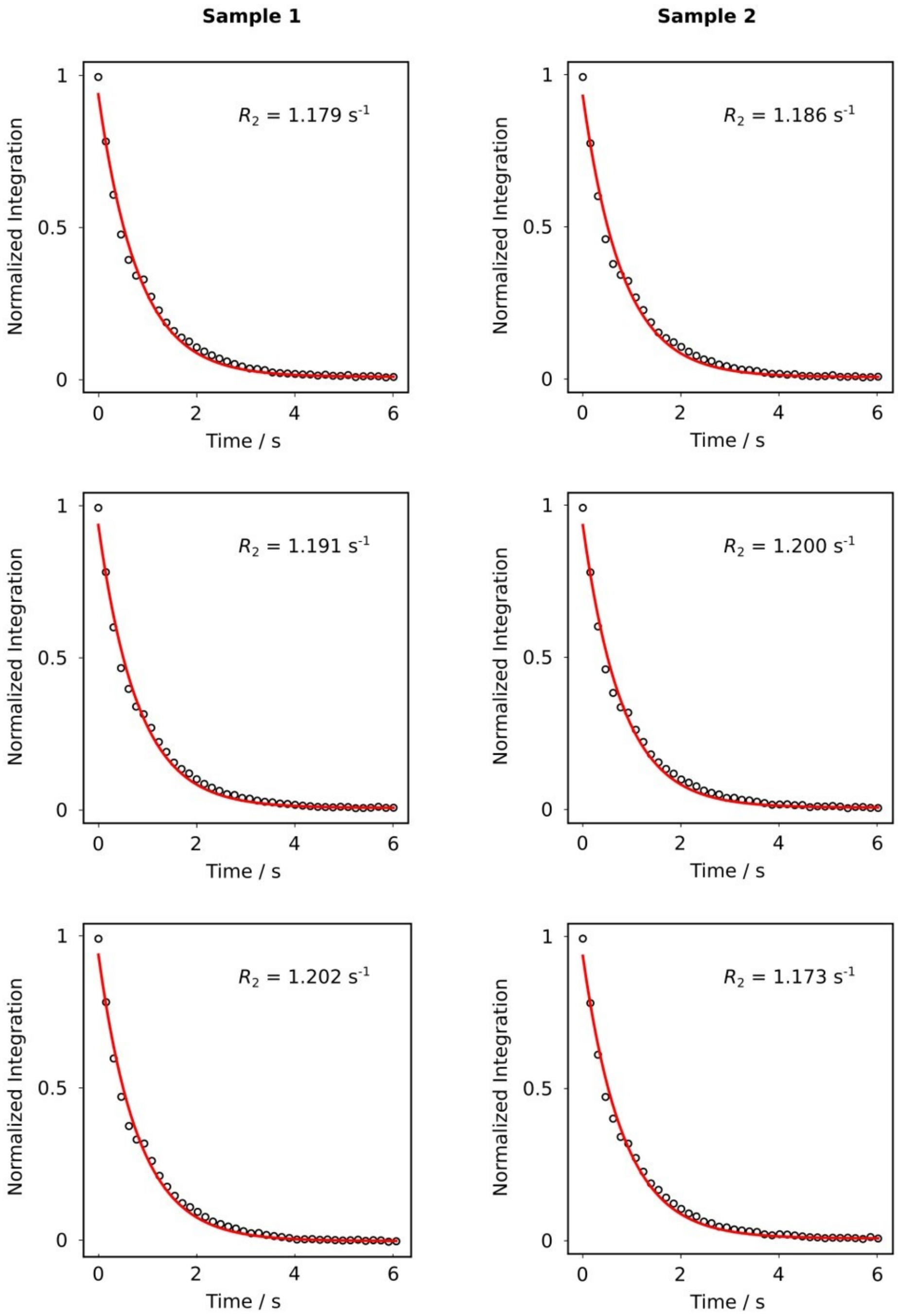

Figure S5: Fitting results of CPMG experiments at low fields (0.85 mT), R_2 = 1.19 ± 0.01 s^{-1}. Two samples consisted of 0.5 mM precatalyst, 5 mM 5-fluoropyridine-3-carboximidamide hydrochloride, and 5 mM dimethyl sulfoxide in methanol. Three measurements were conducted per sample. CPMG trains include 40 π pulses and τ_{CPMG} = 80 ms.

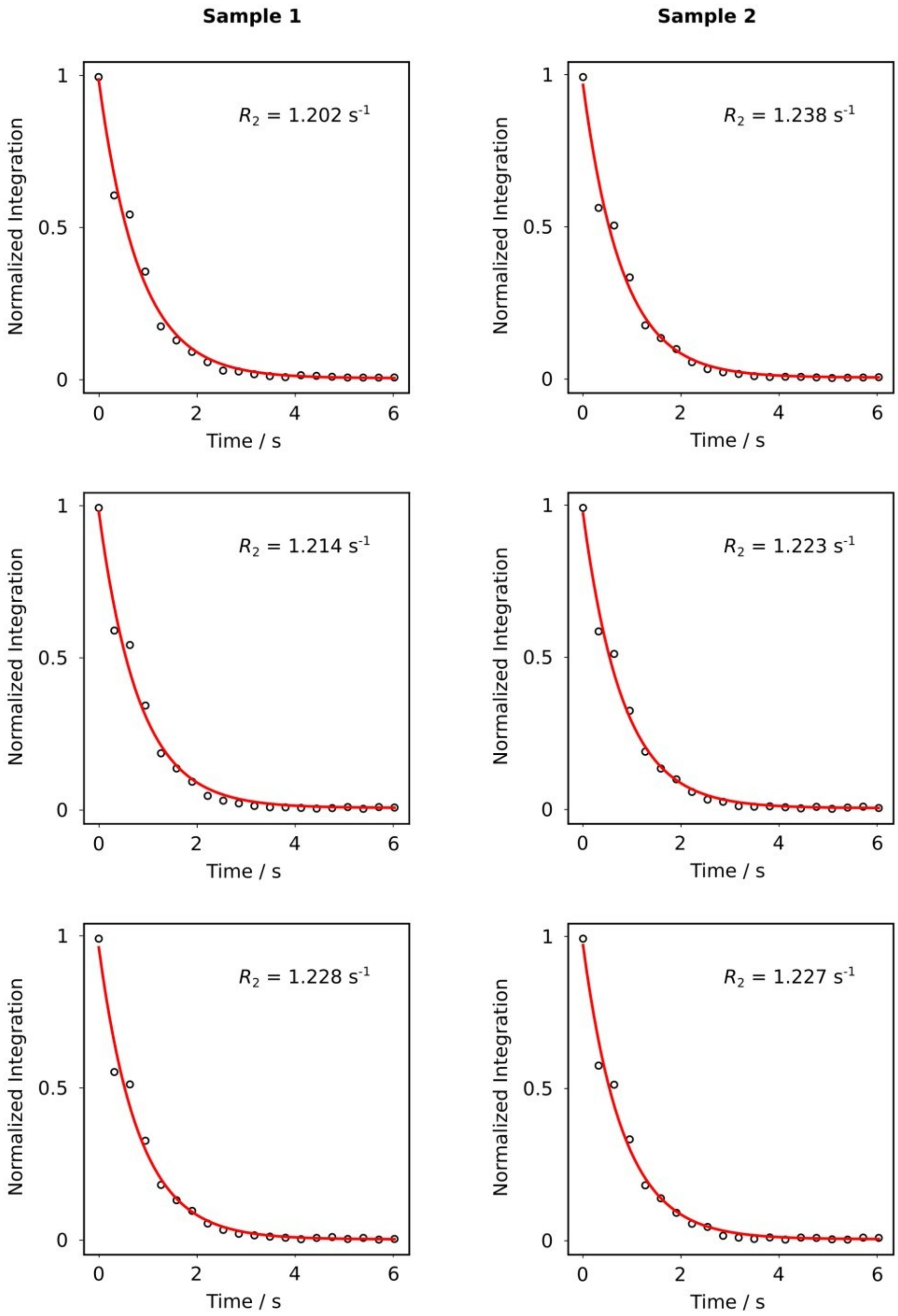

Figure S6: Fitting results of CPMG experiments at low fields (0.85 mT), $R_2 = 1.22 \pm 0.01$ s^{-1}. Two samples consisted of 0.5 mM precatalyst, 5 mM 5-fluoropyridine-3-carboximidamide hydrochloride, and 5 mM dimethyl sulfoxide in methanol. Three measurements were conducted per sample. CPMG trains include 20 π pulses and τ_{CPMG} = 160 ms.

Exchange Spectroscopy

Exchange rates between free and catalyst bound substrate molecules were determined using an Exchange Spectroscopy (EXSY) NMR pulse sequence. In this pulse sequence, free substrate signals were selectively excited. The relative intensity of free substrate signals was measured as a function of the exchange time, which was selected between 10 and 80 ms. Signals were fitted using a two-site exchange model,

$$\frac{d[L_{bound}]}{dt} = -k_d[L_{bound}] + k_{a,app}[L_{free}] \quad (1)$$

$$\frac{d[L_{free}]}{dt} = k_d[L_{bound}] - k_{a,app}[L_{free}] \quad (2)$$

$$k_{ex} = k_d + k_{a,app} \quad (3)$$

Here, [*L*] is the concentration of free or catalyst bound substrate moledule, k_d is the dissociation rate constant, $k_{a,app}$ is the apparent association rate constant, and k_{ex} is the exchange rate of the substrate molecules. The ratio $K = k_d / k_{a,app}$ was independently determined from the integration of the signals of bound and free substrate in one-dimensional spectra of the reaction mixture. This ratio was used as a known quantity in the fit.

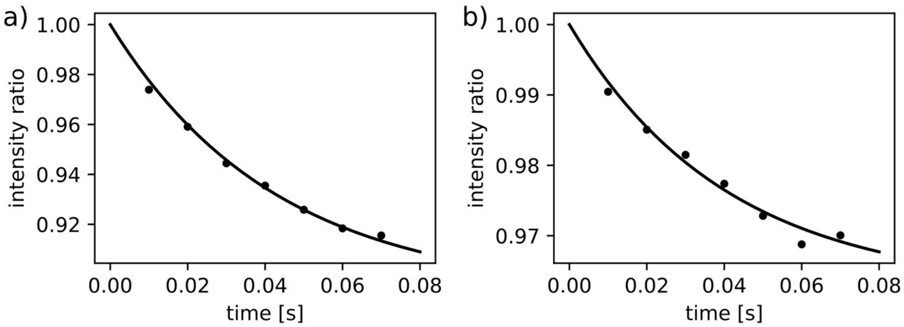

Figure S7: Concentration fractions of free substrate molecules that are determined using a series of EXSY type experiments with various mixing periods from 10 ms to 80 ms after the selective excitation of free substrate molecules. These experiments were conducted for two different ligand concentrations, a) 5 mM and b) 15 mM. In (a), k_{ex} = 23.0 ± 4.0 s^{-1}, $k_{a,app}$ = 2.52 ± 0.04 s^{-1} and k_d = 21 ± 4 s^{-1}. In (b), k_{ex} = 24.6 ± 1.3 s^{-1}, $k_{a,app}$ = 0.923 ± 0.034 s^{-1} and k_d = 23.7 ± 1.4 s^{-1}.
